# Stimuli-Responsive Piezoelectric Scaffold as an Advanced *In Vitro* Platform to Drive Neural Tissue Regeneration

**DOI:** 10.1021/acsomega.6c02450

**Published:** 2026-07-01

**Authors:** Federica Arienti, Noemi Ravaglia, Giorgio Luciano, Diana Pacheco, Pietro Galizia, Carlo Baldisserri, Maurizio Vignolo, Elisa Mercadelli, Tatiana M.F. Patrício, Sabrina Angelini, Monica Montesi, Silvia Panseri

**Affiliations:** † 9327 Institute of Science, Technology and Sustainability for Ceramics (ISSMC), National Research Council of Italy (CNR), Via Granarolo 64, Faenza 48018, Italy; ‡ Department of Pharmacy and Biotechnology-FaBiT, 9296 Alma Mater Studiorum University of Bologna, Via Irnerio 48, Bologna 40126, Italy; § Department of Neuroscience, Imaging and Clinical Science, University of Studies “G. D’Annunzio”, Chieti 66100, Italy; ∥ 458046 Institute of Chemical Sciences and Technologies “Giulio Natta” (SCITEC), National Research Council of Italy, Via De Marini 6, Genova 16149, Italy; ⊥ Centre for Rapid and Sustainable Product Development (CDRSP), 37829 Polytechnic Institute of Leiria, Marinha Grande 2430-028, Portugal; # Coimbra Chemistry Centre-Institute of Molecular Sciences (CQC-IMS), Department of Chemistry, University of Coimbra, Coimbra 3004-535, Portugal; ∇ SeaPower - Association for the Development of the Sea Economy, Industrial Park of Figueira da Foz R. Acácias n.° 40 − A, Figueira da Foz 3090-380, Portugal; □ Clinical Pharmacology Unit, IRCCS Azienda Ospedaliero-Universitaria di Bologna, Via Giuseppe Massarenti 9, Bologna 40138, Italy

## Abstract

Developing physiologically relevant *in vitro* models of the human central nervous system (CNS) remains a significant challenge for neuroscience and translational research. Traditional 2D cultures and animal models often fail to capture the structural, biochemical, and functional complexity of neural tissues, limiting their predictive value for disease modeling and therapeutic testing. Here, we present stimuli-responsive piezoelectric scaffolds as advanced *in vitro* platforms for neural systems. Composed of egg-white proteins and piezoelectric ceramic particles (barium titanate), these scaffolds combine mechanical and structural features of the central nervous system extracellular matrix with autonomous electrical cue generation through mechanical stimulation. Physicochemical characterization demonstrated tunable porosity, mechanical strength, and controlled degradation, while biological evaluation using SH-SY5Y neural cells showed high viability, proliferation, and differentiation. Preliminary assessments suggest that scaffold polarization could play a role in enabling localized bioelectric modulation through remotely applied ultrasound that deforms the piezoelectric particles. By integrating structural and electrical responsiveness, these scaffolds may represent a versatile platform for investigating cell–cell and cell-matrix interactions, mechanotransduction, and bioelectric phenomena in CNS *in vitro* models, potentially offering a scalable and physiologically relevant tool for disease modeling and neuroengineering applications.

## Graphical abstract

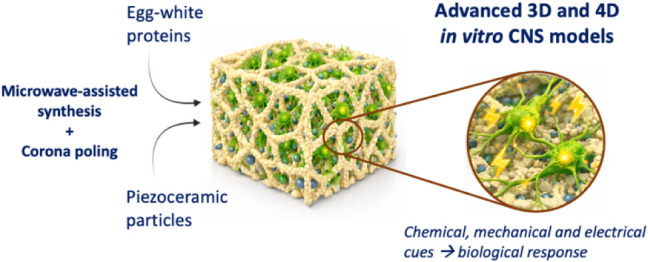

## 1 Introduction

The development of physiologically relevant *in vitro* models of the human central nervous system (CNS) remains a critical challenge for neuroscience and translational research. Traditional two-dimensional (2D) cultures and animal models often fail to capture the structural, biochemical, and functional complexity of human neural tissues, limiting the development of novel clinical approaches and therapeutic screening.^1–3^ This gap is particularly acute in neurodegenerative disorders such as Alzheimer’s disease, Parkinson’s disease, and amyotrophic lateral sclerosis (ALS), as well as in CNS regeneration after trauma, where dynamic, multiscale interactions among neurons, glia, and the extracellular matrix (ECM) are key determinants of disease progression and repair.^4–6^

Human-induced pluripotent stem cell (hiPSC)-derived 3D cultures, including organoids and coculture systems, have advanced our ability to mimic aspects of CNS architecture and function.^7–11^

In parallel, scaffold-based 3D CNS models have emerged as a complementary and increasingly important strategy, in which biomaterial architectures (e.g., hydrogels, electrospun fibers, printed constructs, and porous scaffolds) are used to reproduce key extracellular matrix (ECM)-like physical and biochemical cues.^12–15^ Recent studies have demonstrated that engineered neural scaffolds can improve reproducibility, spatial organization, and microenvironmental regulation compared with spontaneous self-assembled systems, while also allowing more precise tuning of matrix stiffness, porosity, topography, and bioactive signaling pathways.^12,16^

Despite these advances, current models still struggle to replicate cell–cell and cell–matrix interactions, mechanical cues, and bioelectric signals, which are crucial for regulating neuronal and glial behavior. Bioelectricity, arising from ion channel activity and membrane potentials, plays a fundamental role in proliferation, migration, differentiation, and intercellular communication.^17,18^ Despite extensive studies in 2D systems, understanding bioelectrical phenomena in biomimetic 3D environments remains limited. Indeed, most currently available 3D neural models either lack electrically responsive properties or require externally applied stimulation systems, limiting their ability to reproduce the dynamic electromechanical feedback naturally occurring in the CNS microenvironment.^19^ Although electrical stimulation (ES) has been widely used to probe neural function and modulate excitability,^20,21^ conventional ES relies on invasive electrodes or external fields, which can disrupt physiological conditions and limit long-term studies. Electroactive biomaterials have emerged as a promising alternative, delivering electrical or electromechanical cues directly at the cell–material interface.^18,22^ Yet, most platforms remain confined to 2D cultures and depend on external power sources, failing to reproduce the self-regulated bioelectrical environment of native CNS tissues. In addition, several reported electroactive neural scaffolds are often based on synthetic polymers or conductive nanomaterials, which may present limitations in terms of biodegradability, long-term biocompatibility, or biomimetic ECM composition.^23,24^

Piezoelectric biomaterials, defined as materials that generate electrical charge under mechanical stress due to lattice polarization, and vice versa, offer a compelling solution for next-generation CNS models. These materials generate localized electrical signals in response to mechanical deformation, enabling autonomous bioelectrical stimulation without wired electrodes.^25–27^ When integrated into 3D scaffolds, piezoelectric systems support the development of time-responsive (so-called 4D) *in vitro* platforms that evolve over time in response to mechanical forces (e.g., ultrasound application), providing a physiologically relevant mimic of CNS microenvironment.^28–30^ Although piezoelectric scaffolds have been investigated in bone and peripheral nerve engineering, their application in biomimetic 3D CNS *in vitro* models remains relatively limited and less extensively explored, particularly when combined with naturally derived biomaterials.^17,31^ To our knowledge, this is among the first studies to integrate naturally derived egg-based biomaterials with piezoelectric barium titanate particles (BaTiO_3_, BTO) into a 3D neural scaffold specifically designed to provide a CNS-relevant microenvironment. Here, we report the development and characterization of 3D piezoelectric scaffolds, conceived as a platform with the potential to support biomimetic, dynamically responsive CNS models. Egg-derived biomaterials provide an abundant, biocompatible source of proteins that support ECM-like organization, while BTO confers robust piezoelectric properties.^32,33^ Physicochemical, mechanical, and biological characterization of the developed scaffolds was performed to assess their structural properties and *in vitro* behavior.

## 2 Experimental Section

### 2.1 Piezoelectric Scaffolds Synthesis

The egg white proteins (EWP, 1 kg bricks, Eurovo Srl, Italy) were allowed to reach laboratory temperature and were prefoamed using a balloon whisk in a Nalgene beaker. Barium titanate powders (BTO, 99% purity; average particle size ≈700 nm, Merck Sigma-Aldrich), which showed a tetragonal phase and submicrometric particle size (Figure SI 1), were employed as the starting piezoelectric active phase.

Hydroxyapatite (HA) powder was synthesized via wet chemical precipitation using calcium oxide (CaO) (≥99.99% trace metals basis, Merck Sigma-Aldrich, Italy) and ortho-phosphoric acid (H_3_PO_4_) (85%, analytical grade, Merck Sigma-Aldrich, Italy) as precursors, following the protocol described by Dorozhkin,^34,35^ where they were mixed in stoichiometric proportions. The precursor solutions were combined in a flask under continuous magnetic stirring, and the resulting suspension was aged overnight to allow complete precipitation and particle ripening. The precipitate was subsequently collected by vacuum filtration through Whatman filter paper, washed with deionized water to remove ionic impurities, and dried in an oven until a constant weight was achieved. The HA phase was selected for its known foaming behavior under microwave treatment, which provides mechanical reinforcement to the scaffold. In addition, its lower microwave absorption compared to BTO helps limit the temperature increase during processing, thereby preventing thermal degradation of the EWP component and preserving the structural integrity of the composite.

HA and BTO particles were gently embedded into the foam to avoid agglomerations, reaching final EWP:HA:BTO ratios of 6:3:1.5; 6:3:0.3; 1:1:0.1; 1:1:0.5; and 2:1:1. The resulting composite mixtures were cast into silicone molds of two different geometries: 5.0 mm in diameter and 1.5 mm in height for scaffold porosity and mechanical property evaluations, degradation analysis, FTIR, and biological assays; and 10 mm in diameter and 1.5 mm in height for piezoelectric characterization. The samples were then subjected to microwave-assisted heat treatment for sintering—2 min at 700 W for the first two and 4 min at 450 W for the others. During this process, secondary foaming occurred as a result of the microwave-induced expansion.

### 2.2 FTIR Analysis

The ATR-FTIR measurements were carried out on a PerkinElmer Spectrum 2 spectrometer, using 16 accumulations over the 4000–400 cm^–1^ range with a spectral resolution of 0.5 cm^–1^. Prior to analysis, the liquid EWP was converted into a solid by microwave treatment, applying the same protocol adopted for the composite samples. A small aliquot of EWP was deposited on a plate and exposed to microwave irradiation to obtain a dried protein phase, while the remaining starting materials were examined as powders together with the composites, which underwent microwave processing to achieve sintering and to remove the egg-derived aqueous component.

### 2.3 Scaffold Porosity Evaluation

The porosity of every scaffold was first evaluated qualitatively using scanning electron microscopy (SEM) and subsequently also quantitatively using X-ray microcomputed tomography (micro-CT) for the selected EWP:HA:BTO 6:3:1.5 scaffold.

For SEM analysis, the scaffolds were sputter-coated with gold using a Polaron Sputter Coater E5100 and examined at an accelerating voltage of 15 kV under high vacuum (Hitachi TM3000 benchtop SEM), *n* = 2. The microstructure of EWP:HA:BTO 6:3:1.5 and EWP:HA:BTO 6:3:0.3 samples was analyzed with an energy-dispersive X-ray (EDX) detector, *n* = 2.

Micro-CT was then employed to perform a more in-depth analysis of the three-dimensional pore architecture. To enhance scaffold imaging, phosphotungstic acid (PTA) (Sigma-Aldrich, Portugal) was used as a contrasting agent. The staining was performed according to the adapted protocol of Kwon et al.^36^ Therefore, a solution of 1% (w/v) PTA solution was prepared with distilled water. Each scaffold was immersed into 2 mL of PTA staining solution, and the reaction occurred at room temperature overnight. After the staining, the scaffold was washed with distilled water and air-dried for further analysis. Digital radiographs were acquired with a micro-CT scanner (Bruker, SkyScan 1172, USA) by rotating the sample over 180° with a fixed 0.9° step, a source voltage of 50 kV, and a source current of 800 μA. Experimental conditions were set to optimize acquisition time and achieve the best image contrast, with a pixel size resolution of 7.7 μm and an average of three radiographs per position. Slice reconstruction of the raw scanned data was carried out with NRecon 1.6.3 software. During the reconstruction, the value of ring artifact reduction was set to 11%, smoothing to 2%, and the value of beam-hardening correction to 30%. 3D model visualization was obtained using the CTVox software (Bruker). 3D analysis (total porosity, pore size, and distribution) was performed with the CT-Analyzer v1.20 software, *n* = 3.

### 2.4 Dynamic Mechanical Analysis

Dynamic Mechanical Analysis (DMA) was performed on EWP:HA:BTO scaffolds (6:3:1.5 and 6:3:0.3 formulations) using a Q800 dynamic mechanical analyzer (Q800 V20.26 Build 45, TA Instruments), equipped with a compression clamp, to determine the compressive mechanical properties (i.e., Young’s modulus). Samples (5.0 mm in diameter, 1.5 mm in height) were preconditioned by overnight immersion in PBS 1X (Gibco) at 37 °C. All measurements were subsequently performed at 37 °C under PBS 1X immersion conditions to replicate physiological-like environments. Before testing, the instrument was calibrated according to the manufacturer’s instructions. The compressive Young’s modulus was determined from stress–strain curves obtained under compressive loading. In particular, a stress–strain test was performed, consisting of an isothermal equilibration period of 5 min at 37 °C, followed by a force-controlled ramp at a rate of 0.1 N/min from 0 to 1 N, then a force ramp rate of 0.5 N/min from 1 to 5 N, and ending with a force ramp rate of 1 N/min from 5 to 18 N. Prior to starting the measurement, a preload force of 10–4 N was always applied to the sample to ensure the entire scaffold surface was properly in contact with the compression plate. The Young’s modulus was calculated as the slope of the linear region of the stress–strain curve (0–10% strain) (*n* = 4).

### 2.5 Degradation Test

A degradation test was conducted on the selected EWP:HA:BTO 6:3:1.5 scaffold to evaluate scaffold stability over time under physiological conditions. Dry scaffolds were individually immersed in 500 μL of PBS 1X in a 48-well plate and incubated at 37 °C under static conditions. At predetermined time points—0 d, 1 d, 3 d, 7 d, 10 d, 14 d, 18 d, 21 d, 24 d, 30 d, and 35 d—each scaffold was removed from the PBS 1X, carefully dried with filter paper to remove excess liquid, and weighed using an analytical balance with a readability of 0.1 mg (±0.1 mg). The PBS 1X solution was refreshed every 2 days to better simulate medium exchange conditions commonly used in *in vitro* studies.

The degradation percentage (*D*) was calculated as follows:

$$D\;(\%)=\left(\frac{Wi-Wf}{Wi}\right)\times 100$$

where *Wi* is the initial weight immediately after immersion in PBS 1X, and *Wf* is the weight at the selected time point. The data are reported as a percentage with respect to the 0 d time point (*n* = 5).

Finally, digital images of the samples were captured at each time point to allow for a qualitative assessment of the degradation process over time.

A protein quantification assay was conducted on the supernatants collected at each degradation time point, to assess protein release associated with scaffold degradation. Protein content was determined using a Lowry assay (DC Protein Assay Kit , Bio-Rad), following the manufacturer’s protocol. Absorbance was measured at 750 nm with a microplate reader (Multiskan FC, Thermo Scientific), and protein concentrations were calculated through a standard curve generated with bovine serum albumin (BSA, PAA) according to the Bradford method.^37^

### 2.6 Corona Poling and Piezoelectric Testing

EWP:HA:BTO 6:3:1.5 scaffold samples were poled at room temperature using a custom-built corona discharge setup. A voltage of 20 kV was applied for 5 min, with the needle positioned 5 cm from the sample surface. The piezoelectric activity was checked by measuring the d_33_ piezoelectric coefficient using a d_33_ meter (SinoCera, Sinoceramics, Shanghai, China), which applies a periodic force at 110 Hz with an amplitude of 0.25 N over a circular contact area of 2.5 mm in diameter. Prior to the measurements, the instrument was calibrated using a 360 pC/N standard provided by the instrument supplier. Six measurements were performed, three for each poling orientation (positive and negative). The reported d_33_ value represents the mean ± standard deviation of these measurements.

### 2.7 *In Vitro* Biological Study

The SH-SY5Y human neuroblastoma cell line (ATCC CRL-2266) was maintained in complete growth medium (GM) composed of DMEM/F-12 (Dulbecco’s Modified Eagle Medium/Nutrient Mixture F-12, Gibco) supplemented with 10% Fetal Bovine Serum (FBS, Gibco) and 1% Penicillin and Streptomycin solution (pen/strep, 100 U/mL–100 μg/mL, Gibco). All cell cultures were kept at 37 °C under 5% CO_2_ atmosphere conditions and controlled humidity. Cells were harvested through trypsinization, and following centrifugation, the cell count and viability were determined using the Trypan Blue Dye exclusion assay. For the biological evaluation, EWP:HA:BTO 6:3:1.5 scaffolds were sterilized with 70% ethanol, followed by ultraviolet (UV) irradiation for 5 min in PBS 1X under a sterile laminar-flow hood. Subsequently, the samples were preconditioned by overnight incubation in a 48-well plate in complete cell culture medium at 37 °C. The preconditioned scaffolds were then transferred to a new 96-well plate and seeded by carefully dropping 5 μL of cell suspension (6.0 × 10^4^ cells) on the top surface, followed by a 20-min incubation to promote cell adhesion before adding 170 μL of GM. For neuronal differentiation, after 8 days of cell growth in GM, the protocol required the use of DMEM/F-12 containing 2% FBS, 1% pen/strep, and All-Trans Retinoic Acid 10 μM (RA, STEMCELL) for 3 days, followed by DMEM/F-12 containing 1% FBS, 1% pen/strep, RA 10 μM, and Human Recombinant Brain-Derived Neurotrophic Factor 10 ng/mL (BDNF, STEMCELL) for 7 days (differentiation medium, DM). All cell-handling procedures were performed under a biological laminar-flow hood and sterile conditions. The cell medium was replaced every 3 days until the end of the experiment.

#### 2.7.1 Cell Viability and Proliferation Tests

A qualitative LIVE/DEAD assay was performed on days 1, 4, 14, and 18, following the manufacturer’s instructions, to evaluate cell viability as a qualitative analysis. Briefly, cells were washed with PBS 1X and incubated with 1.3 μM Calcein AM and 4 μM Ethidium homodimer-1 for 15 min at 37 °C and 5% CO_2_. Images of live cells stained in green and dead cells stained in red were acquired by using an inverted Ti-E fluorescent microscope (Nikon). One sample for each time point was analyzed (*n* = 1).

Thiazolyl Blue Tetrazolium Bromide (MTT) assay was employed to carry out a quantitative evaluation of cell viability and proliferation over time at days 1, 4, 11, and 18 of culture, according to the manufacturer’s instructions. Briefly, at each time point, every sample was incubated with 10% (v/v) MTT solution (5 mg/mL, Merck) for 2 h at 37 °C, 5% CO_2_ atmosphere and controlled humidity conditions. The metabolically active cells reacted with the tetrazolium salt in the MTT reagent, resulting in the formation of formazan crystals. Then, scaffolds were transferred to a tube containing 300 μL of dimethyl sulfoxide (DMSO, Sigma-Aldrich) to dissolve the insoluble formazan crystals derived from MTT conversion. After incubating for 15 min, the supernatants were collected and analyzed with a UV–visible spectrophotometer (Multiskan FC, Thermo Scientific) by measuring the absorbance at 570 nm. Three samples for each time point (days 1, 4, 11, 18) were analyzed (*n* = 3), and the corresponding blank average (scaffold without any cells seeded) was subtracted from each measurement.

Both cell viability and proliferation tests (LIVE/DEAD assay and MTT test) were performed in the GM and DM.

#### 2.7.2 Cell Morphology Evaluation

The morphology of SH-SY5Y cells on the EWP:HA:BTO 6:3:1.5 scaffold was visualized by fluorescent staining of actin filaments and by scanning electron microscopy.

Concerning actin staining, on day 18 of culture, samples were fixed with 4% (w/v) paraformaldehyde (PFA, Merck KGaA) for 15 min, followed by cell membrane permeabilization with 0.1% (v/v) Triton X-100 (Sigma-Aldrich) in PBS 1X for 15 min at room temperature. Actin filaments were stained using Actin Red 555 ReadyProbes Reagent (Invitrogen) for 30 min, and cell nuclei were counterstained with DAPI (600 nM, Invitrogen) in PBS 1X for 7 min. After one final wash in PBS 1X, fluorescent images were captured using a Nikon inverted Ti-E fluorescence microscope. One sample for each condition (GM and DM) was analyzed (*n* = 1).

The qualitative cell morphology analysis performed by SEM was also conducted on day 18 of culture. Briefly, the samples were washed in 0.1 M Sodium Cacodylate Buffer (pH 7.4, Sigma-Aldrich) and fixed in a 2.5% Glutaraldehyde (Merck KGaA) solution in 0.1 M Sodium Cacodylate Buffer for 2 h at 4 °C. Subsequently, they were washed once with 0.1 M cacodylate buffer (pH 7.4) for 5 min and twice with Milli-Q water for 10 min. Finally, the samples were frozen at −20 °C, freeze-dried, sputter-coated with gold as described in Section 2.2, and analyzed at an accelerating voltage of 10 kV under high vacuum (FEI Quanta200, ESEM). One sample for each condition (GM and DM) was analyzed (*n* = 1).

#### 2.7.3 Immunofluorescence Analysis

Neuronal differentiation was assessed by immunofluorescence staining of β-III tubulin (TUBB3, TUJ1 clone, STEMCELL), an early neuronal cytoskeletal marker, in cells cultured on the EWP:HA:BTO 6:3:1.5 scaffolds for 18 days in DM, with cells maintained on the same scaffolds in growth medium (GM) used as controls.

Briefly, the samples were fixed in 4% (w/v) PFA for 15 min, then washed 3 times in PBS 1X for 5 min, followed by blocking in 1% BSA + 10% Normal Goat Serum (NGS, Euroclone, Italy) in PBS 1X for 30 min at room temperature under slow agitation. The cell membranes were permeabilized in PBS 1X with 0.1% (v/v) Triton X-100 for 15 min at room temperature. Subsequently, after three more washes in PBS 1X, the primary antibodies against β-III tubulin (1:2000, mouse 60052, Stem Cell) in 1% NGS in PBS 1X were added to the samples and left to incubate overnight at 4 °C in a humidity chamber.

A donkey anti-mouse IgG antibody (1:500, Alexa Fluor 555 donkey antimouse IgG H and L, Invitrogen) was used as the secondary antibody in 1% NGS in PBS 1X for 1-h incubation at room temperature in the dark. For cell nucleus detection, DAPI staining (600 nM in PBS 1X, 10 min at room temperature) was used. After one final wash in PBS 1X, the scaffolds were observed with a Nikon Inverted Ti-E fluorescent microscope. One sample for each condition (GM and DM) was analyzed (*n* = 1).

### 2.8 Statistical Analysis

Results of pore size distribution were reported as mean ± standard deviation (SD). All the other results were plotted as mean ± standard error of the mean (SEM), and statistical analyses were performed by GraphPad Prism Software (Version 8.0). Young’s moduli were analyzed by one-way analysis of variance (one-way ANOVA), followed by Tukey’s Multiple Comparisons test. SH-SY5Y proliferation data were analyzed by two-way analysis of variance (two-way ANOVA), followed by Sidak’s Multiple Comparisons test. Statistically significant differences, if any, are reported in the graphs: *p ≤ 0.05, **p ≤ 0.01, ***p ≤ 0.001, and ****p ≤ 0.0001.

## 3 Results and Discussion

### 3.1 Physicochemical and Structural Characterization of Scaffolds

#### 3.1.1 Synthesis and ATR-FTIR Characterization of EWP:HA:BTO Composite Scaffolds

All scaffold formulations were successfully synthesized across all investigated compositions (i.e., EWP:HA:BTO ratios of 6:3:1.5; 6:3:0.3; 1:1:0.1; 1:1:0.5; and 2:1:1), yielding homogeneous and structurally coherent composites. The incorporation of HA into protein-based composites offers established biocompatibility and favorable foaming properties under microwave treatment.^38^ HA was selected based on prior experience with its microwave responsiveness, which produces lower heating rates compared to high-absorption ceramics such as barium titanate, thus mitigating thermal damage to proteins.^39^ In addition, HA has been widely employed as a reinforcing phase in composite scaffolds owing to its chemical stability and compatibility with biological environments, contributing to the fabrication of structurally stable porous constructs.^40^

The embedding of BTO particles into the prefoamed EWP matrices proceeded without observable agglomeration, and the subsequent microwave-assisted heat treatment produced stable scaffolds with consistent dimensions and reproducible microstructural features suitable for subsequent evaluations.

Representative macroscopic images of each scaffold formulation are shown in Figure SI 2. The scaffolds maintained their overall integrity after fabrication and could be readily handled without structural damage, enabling their use in subsequent experimental evaluations.

The absence of visible particle aggregation is particularly relevant, as homogeneous BTO dispersion is expected to promote a more uniform distribution of piezoelectric domains within the scaffold and more reproducible electromechanical behavior. Figure 1(A) shows the ATR-FTIR spectra of the precursor phases (HA, microwave-treated EWP, and BTO), while Figure 1(B) reports the spectrum of the ternary composite. The precursor spectra exhibit distinct fingerprints that serve as references for monitoring phase evolution in the scaffold. HA displays the typical PO_4_^3–^ bands (ν_1_, ν_2_, ν_3_, ν_4_), with ν_3_ and ν_4_ modes at ∼1020 and 560 cm^–1^ used as markers of the hydroxyapatite phase, while weak OH (∼3570 cm^–1^ stretching OH), and carbonate (∼1400 cm^‑1^) features indicate limited surface hydration and substitution.^35,41^ The EWP pattern is dominated by a broad O–H/N–H stretching envelope (2850–3750 cm^–1^) and by amide I–III bands between 1500 and 1700 cm^–1^, which define the protein fingerprint and provide a spectral window to assess the retention of the organic phase after processing.^42,43^ BTO exhibits the characteristic low-wavenumber Ti–O absorptions of TiO_6_ octahedra, with only minor high-frequency contributions attributed to trace residual species.^44,45^

**Figure 1:**
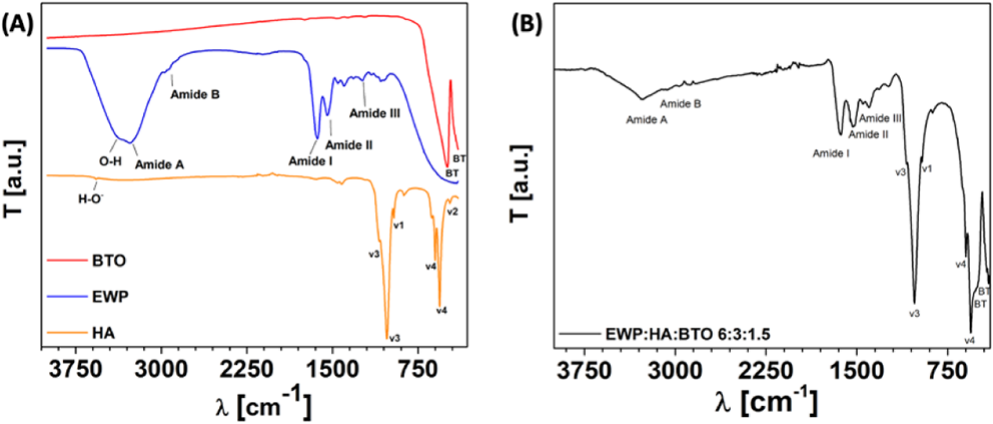
ATR-FTIR analysis: (A) Spectra of the precursor phases (HA, EWP, and BTO). (B) Spectrum of the ternary composite.

The ATR-FTIR spectrum of the EWP:HA:BTO composite in Figure 1(B) retains the HA marker bands at ∼1022 and 560 cm^–1^, confirming effective hydroxyapatite incorporation. A broad band around 3310 cm^–1^ and residual signals near 1630–1550 cm^–1^ assigned to amide I–II vibrations of EWP, indicate that a portion of the proteinaceous component remains embedded within the porous hybrid scaffold after processing. This conclusion is based on the persistence of these amide bands, although ATR-FTIR in this configuration does not allow reliable quantification of the residual EWP fraction. This observation is particularly relevant because the retention of protein-derived functional groups may contribute to maintaining a biologically favorable microenvironment and support subsequent cell–material interactions. Furthermore, the simultaneous presence of characteristic signals from both the inorganic (HA and BTO) and organic (EWP) phases confirms the successful formation of a hybrid composite scaffold without evidence of major phase degradation.

#### 3.1.2 Morphological and Mechanical Properties of Scaffolds

SEM analysis revealed distinct morphological differences among the five scaffold formulations. Scaffolds with higher EWP:HA ratios exhibited greater porosity. Specifically, the EWP:HA:BTO ratios of 6:3:1.5 and 6:3:0.3 displayed a more open and interconnected porous structure compared to the more compact formulations (1:1:0.1, 1:1:0.5, and 2:1:1) (Figure 2A). Given their limited porosity, the latter formulations were not considered suitable candidates for neural tissue engineering applications and were therefore not investigated further. In fact, porosity is a key structural parameter in three-dimensional scaffolds, as it directly influences cell adhesion, migration, nutrient diffusion, and waste exchange, ultimately affecting tissue development and maturation.^46^ An interconnected porous architecture is particularly critical for neural tissue engineering, where cellular colonization and network formation rely on both adequate pore size and spatial continuity.^47,48^ To further investigate the distribution of the inorganic phases, EDX elemental mapping was performed, revealing a homogeneous distribution of both HA and BTO particles throughout the EWP:HA:BTO 6:3:1.5 and EWP:HA:BTO 6:3:0.3 scaffolds, respectively (Figure 2B,C). The absence of evident particle aggregation suggests effective incorporation of the ceramic phases within the protein matrix, an important prerequisite for achieving homogeneous mechanical properties and, in the case of BTO-containing systems, a uniform piezoelectric response. Based on these observations, mechanical properties were assessed on EWP:HA:BTO 6:3:1.5 and EWP:HA:BTO 6:3:0.3 samples. Young’s modulus measurements indicated values within the range of soft tissues (i.e., 6.17 ± 0.30 kPa and 8.17 ± 1.07 kPa for EWP:HA:BTO 6:3:1.5 and 6:3:0.3, respectively), including central nervous system tissue, with no statistically significant difference between the two scaffolds (Figure 2D).^49,50^ Considering the combined results of porosity, inorganic phase distribution, and mechanical properties, both formulations emerged as suitable candidates for neural tissue engineering applications. However, for subsequent investigations, the EWP:HA:BTO 6:3:1.5 scaffold was selected as the most promising formulation because its higher BTO content was expected to enhance the piezoelectric response, which represents the primary functional objective of the present study.

**Figure 2:**
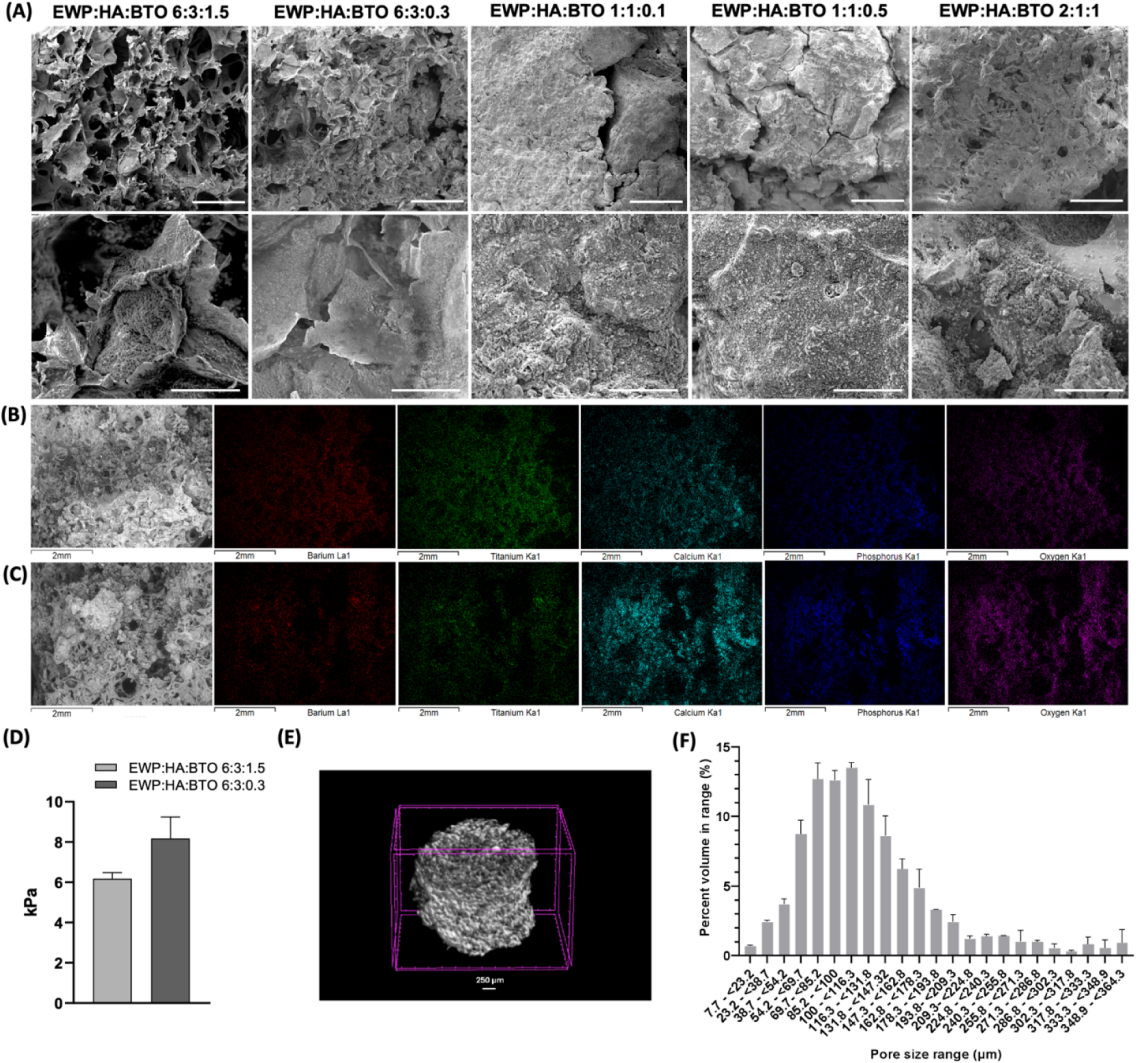
(A) Representative SEM images of EWP:HA:BTO scaffolds prepared at different weight ratios. Low-magnification images (top row) highlight the scaffold porosity, showing a more open and interconnected porous structure in the first two formulations (EWP:HA:BTO 6:3:1.5 and 6:3:0.3) compared with the more compact formulations (EWP:HA:BTO 1:1:0.1, 1:1:0.5, and 2:1:1); scale bars: 500 μm. High-magnification images (bottom row) show the distribution of BTO particles throughout the scaffold matrix; scale bars: 50 μm. *n* = 2. SEM images and corresponding EDX elemental maps of EWP:HA:BTO 6:3:1.5 (B) and EWP:HA:BTO 6:3:0.3 (C) scaffolds. Elemental mapping was performed to investigate the distribution of the inorganic phases within the scaffold matrix. The distribution of titanium (Ti), barium (Ba), calcium (Ca), phosphorus (P), and oxygen (O) is shown, confirming the presence and spatial distribution of the BTO and HA phases. (D) Dynamic mechanical analysis scaffold characterization (Young’s modulus). Results presented are mean ± SEM, n = 4. (E) Three-dimensional computed tomography model of the EWP:HA:BTO 6:3:1.5 sample. (F) Pore size distribution of the EWP:HA:BTO 6:3:1.5 scaffold. Results presented are mean ± SD, *n* = 3.

A more detailed assessment of its 3D architecture was then performed using micro-CT. The 3D micro-CT models of the scaffold (Figure 2E, Video SI 1) revealed a heterogeneous yet interconnected structure, with a total porosity of 77.22 ± 1.32%. This value falls within the range considered suitable for neural tissue engineering. Previous studies have reported that scaffold porosities above 70% facilitate cell infiltration, nutrient diffusion, and neurite extension.^51,52^ Moreover, internal morphology analysis (Figure 2F) showed pore sizes ranging from 7.7 to 364.3 μm. While pores larger than 240 μm represented only 6.7% of the total pore volume, the predominant pore population (38.8%) was distributed within the 69.7–116.3 μm range. This pore size distribution is consistent with values reported to support neural cell attachment, migration, differentiation, and neurite outgrowth, whereas high scaffold porosity favors nutrient transport and cell colonization.^52–54^

#### 3.1.3 Stability and Degradation Behavior of the Sample

The degradation profile of the selected scaffold (EWP:HA:BTO 6:3:1.5, Figure 3A) was assessed in PBS 1X at 37 °C to mimic physiological conditions. The scaffold remained structurally stable for up to 21 days, exhibiting a minimal mass loss of approximately 17.05 ± 7.6% of its initial weight. Beyond this period, the scaffold gradually degraded, achieving complete degradation by day 35 (Figure 3B).

**Figure 3:**
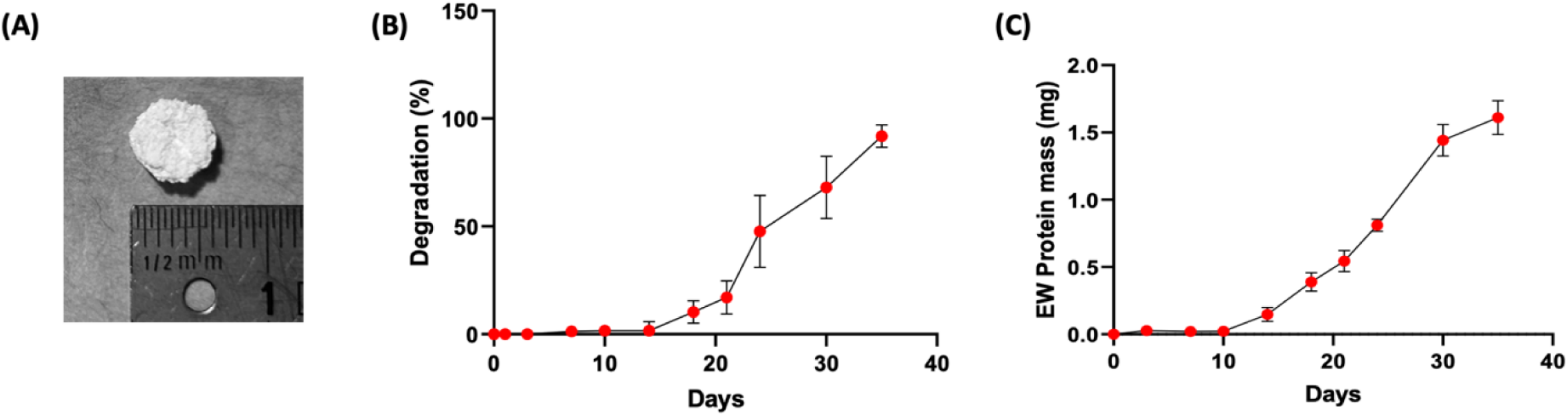
(A) Representative image of the scaffold before testing. (B) Scaffold degradation profile over time, expressed as % weight loss relative to day 0. (C) Protein release from the scaffold over time (in mg), quantified using a Lowry assay.

This degradation time scale is consistent with that reported for protein-based and composite scaffolds designed for soft tissue engineering. From a neural tissue engineering perspective, this degradation window is particularly relevant, as similar time scales have been reported for scaffold-assisted neuronal adhesion, neurite extension, and early network formation in 3D cultures.^55,56^

Quantitative analysis of protein release from the supernatants collected at each time point demonstrated a clear correlation between scaffold degradation and protein release. Initially, the release was minimal, consistent with the structural stability observed on day 21. As degradation progressed beyond this period, protein release increased proportionally, reaching maximal levels concurrent with complete scaffold breakdown on day 35 (Figure 3C). These results indicate that the scaffold provides stable support under physiological conditions for at least 3 weeks, while allowing a controlled and predictable release of protein content as degradation proceeds, supporting its potential for neural tissue engineering applications where temporary structural support and gradual biochemical delivery are desired.

### 3.2 *In Vitro* Biological Evaluation of Scaffolds

The *in vitro* biological performance of the EWP:HA:BTO 6:3:1.5 scaffold was evaluated to determine its suitability as a three-dimensional platform for neural cell culture. Cell viability, morphology, and spatial organization within the scaffolds were assessed to provide a comprehensive understanding of cell–scaffold interactions and behavior within the 3D architecture. Seeded cells exhibited high viability on both day 1 and day 4, as confirmed by Live/Dead staining, which revealed a predominance of live cells over a minimal number of dead cells (Figure 4A,B). High cell viability in 3D scaffolds is a fundamental requirement for neural tissue engineering and has been consistently reported as a primary indicator of scaffold cytocompatibility in biomaterial-based neural culture systems.^57,58^ On day 4, half of the samples were transferred to differentiation medium (DM), while the remaining half were maintained in complete growth medium (GM), and the cultures were maintained up to day 18. Quantitative MTT assay indicated progressive cell proliferation on the scaffolds over time, with no statistically significant differences between GM and DM conditions, suggesting that the scaffold provides a supportive environment for cell survival and growth (Figure 4C). Qualitative morphological analysis revealed the establishment of an extensive cellular network under both conditions, with cells efficiently colonizing the scaffold and forming interconnected structures throughout the 3D matrix, as evidenced by both actin filament staining and SEM imaging (Figure 4D–G). 3D scaffolds are known to enhance cell–cell interactions and promote more physiologically relevant neural-like network formation compared to 2D cultures.^59,60^ To further characterize neuronal differentiation, immunofluorescence staining for β-III tubulin, a well-established early neuronal cytoskeletal marker involved in neurite outgrowth and network formation,^61,62^ was performed. In cells maintained in GM, β-III tubulin expression was primarily confined to the cytoplasm, and cells did not exhibit evident neurite extensions (Figure 4H). In contrast, cells cultured in DM displayed a marked redistribution of β-III tubulin along elongated neuritic processes, consistent with a more mature neuronal morphology (Figure 4I,J; Videos SI 2, 3). This neuritic pattern was accompanied by prominent neurite outgrowth and the formation of initial neural networks, supporting the scaffold’s ability to promote early neuronal differentiation and network assembly within a physiologically relevant 3D environment.

**Figure 4:**
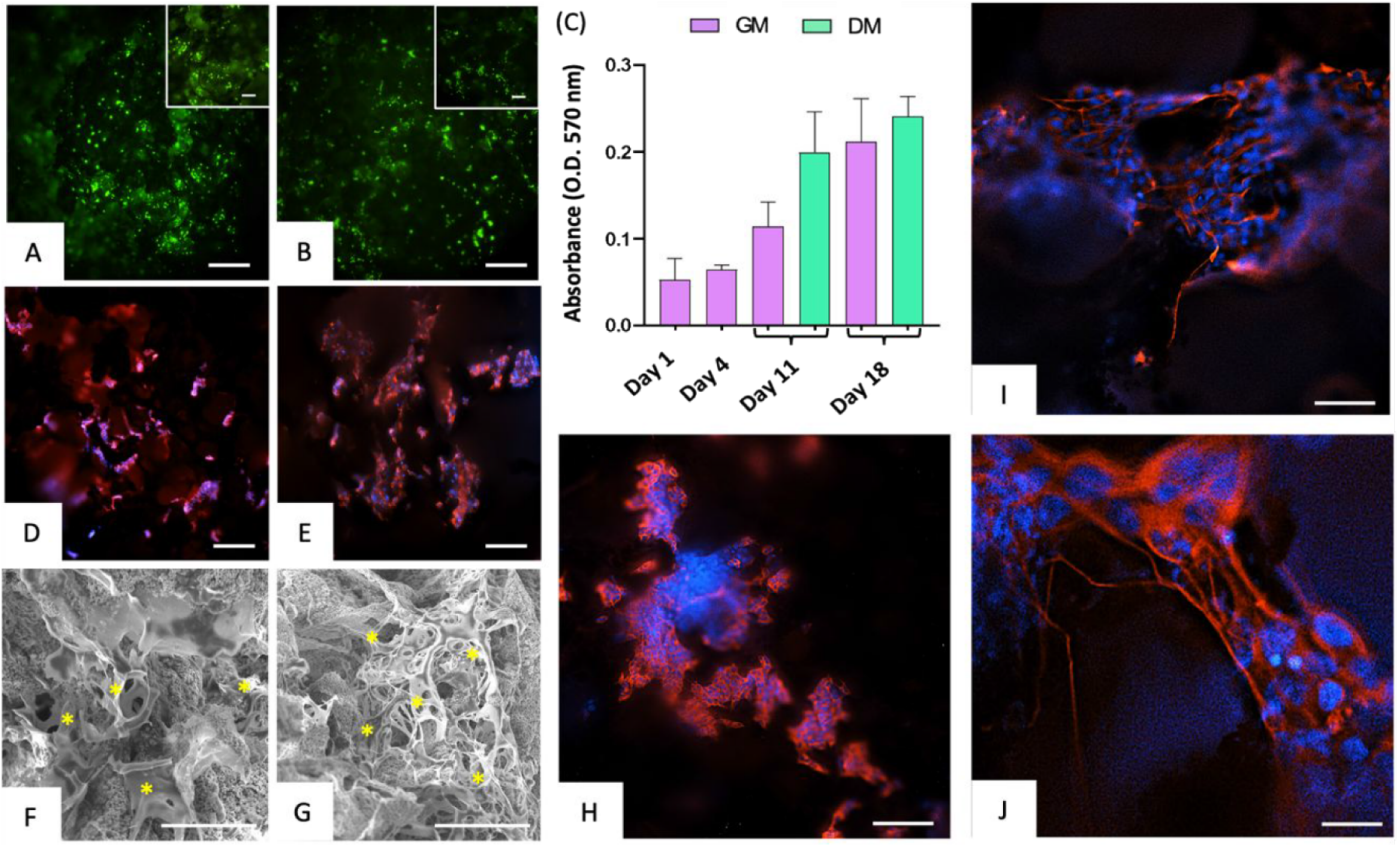
LIVE/DEAD assay to assess cell viability at 1 day (A) and 4 days (B) post-seeding on EWP:HA:BTO 6:3:1.5. Live cells are shown in green; dead cells in red. Scale bars: 500 μm, insets 200 μm. (C) Cell proliferation measured by MTT test after 1, 4, 11, and 18 days of culture in growth medium (GM) and differentiation medium (DM) (mean ± SEM, *n* = 3). (D–G) Morphological cell evaluation after 18 days of culture on EWP:HA:BTO 6:3:1.5 in GM (D–F) or DM (E–G). (D, E) Actin filaments are shown in red; cell nuclei in blue. Scale bars: 100 μm. (F, G) SEM images, with yellow asterisks highlighting the cellular network. Scale bars: 50 μm. Immunofluorescence detection of β-III tubulin in cells cultured for 18 days on EWP:HA:BTO 6:3:1.5 scaffold in GM (H) or DM (I, J) conditions. β-III tubulin is shown in red; cell nuclei in blue. Scale bars: H, I 100 μm; J 50 μm.

### 3.3 Scaffold Polarization and Early Cellular Behavior

As introduced above, piezoelectric biomaterials are increasingly attractive for CNS *in vitro* models because of their ability to transduce mechanical stimuli into local electrical cues, thereby mimicking the electromechanical microenvironment of neural tissues. This capability is particularly relevant, since endogenous electrical signals are known to regulate neuronal adhesion, differentiation, and synaptic activity. However, to elicit a biologically relevant piezoelectric response, an appropriate material polarization step is required to induce stable surface charges and activate the piezoelectric domains.

In this context, the effect of polarization on the bioactivity of the EWP:HA:BTO 6:3:1.5 scaffold was investigated by comparing poled and non-poled samples in a preliminary *in vitro* study. Consistent with literature reports showing that polarization-induced surface charges can modulate cell behavior,^63,64^ cells were seeded on the positively poled surface and compared with non-poled scaffolds. Live/Dead staining at 24 h postseeding showed high cell viability on both surfaces, but notably higher cell density on the poled scaffold (Figure 5A,B). Although the measured d_33_ value was relatively low, it falls within the range reported for highly porous polymer–ceramic composite scaffolds containing BaTiO_3_, where the effective piezoelectric response is often reduced by the compliant matrix and high porosity.^65–67^ In detail, corona poling aligned the dipoles of the BTO particles embedded in the foamed EWP matrix, generating two stable surfaces of opposite polarity and inducing measurable piezoelectric activity, as confirmed by a d_33_ coefficient of 0.7 ± 0.2 pC/N. The observed increase in initial cell density on the poled scaffolds suggests that the polarization treatment may influence early cell–material interactions. The polarization process is well-known to produce long-lived surface charges that can interact with biological systems.^68^ Although the low stiffness and dielectric permittivity of the foamed EWP matrix limit the mechanical coupling and the effective piezoelectric response, the scaffolds were successfully polarized and showed an effective piezoelectric activity that could be compatible with local cellular signals. An order-of-magnitude estimate of the electric field generated under ultrasound can be derived from the linear piezoelectric constitutive equation^69^

D=dσ+εE

where *D* is electric displacement (C/m^2^), *d* is the piezoelectric coefficient (C/N), σ is the mechanical stress (N/m^2^), *ε* is the permittivity at constant stress (F/m), and *E* is the electric field (V/m). In this formulation, *dσ* represents the stress-induced piezoelectric charge contribution, while *εE* represents the charge associated with the internal electric field. Under open-circuit conditions (no external electrical connection), the net free-charge flux is suppressed, and the electric displacement can be approximated as *D* ≈ 0, which yields

|E|≈dσ/ε

**Figure 5:**
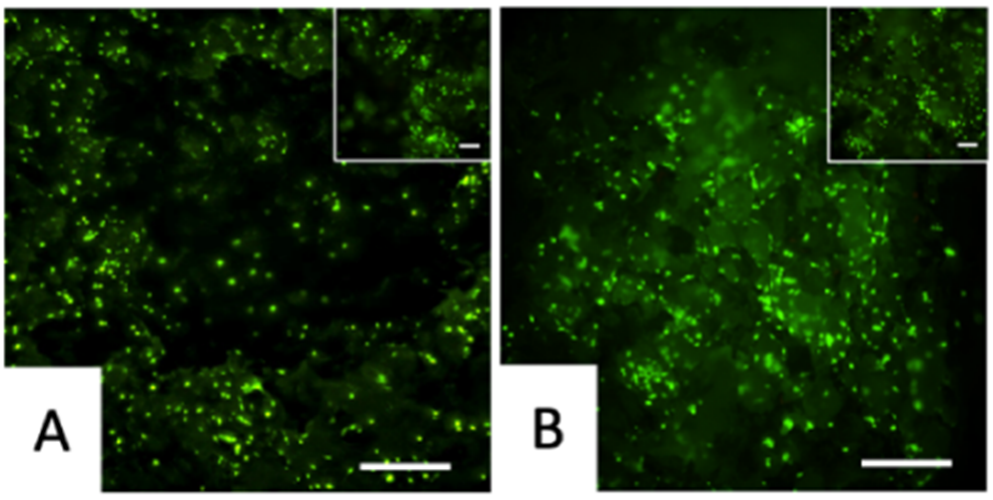
LIVE/DEAD assay to assess cell viability after 24 h postseeding on EWP:HA:BTO 6:3:1.5 non-poled (A) and poled (B) samples. Live cells are shown in green; dead cells in red. Scale bars: 500 μm, inset 200 μm.

Assuming a relative permittivity *ε_r_* in the 150–200 range, the permittivity, *ε* = *ε*_0_ *ε*_*r*_, where *ε*_0_ = 8.854 pF/m. To estimate the mechanically applied stress (*σ*) under ultrasound, the acoustic intensity *I* can be related to the root-mean-square acoustic pressure *(p*_*rms*_ *)* for a plane wave in a fluid medium as^70^

$$I=p_{rms}^{2}/\rho c$$

where *I* is the ultrasound intensity (W/m^2^), *ρ* is the density of the medium (≈1000 kg/m^3^ for water-based media), and *c* is the sound speed (≈1500 m/s in water). Neglecting attenuation and assuming that the acoustic pressure is transferred as an effective stress to the piezoelectric phase, one can set *σ* ≈ *p*_rms_ as a first approximation. Considering ultrasound stimulations generated by a common ultrasonic processor (Vibra-Cell VCX 130, Sonics & Materials Inc.) equipped with a 6 mm diameter probe, operating at 20% amplitude and a frequency of 20 kHz, the stimulation intensity applied to the scaffold was estimated as *I* ≈ 9 kW/m^2^.^71^ This corresponds to σ on the order of 10^2^ kPa. Therefore, the resulting electric field magnitude is expected to be on the order of a few tens of V/m. While this is a simplified estimate, it indicates that the scaffold may generate local electrical stimuli under relevant mechanical inputs. Future work will address the temporal evolution of d_33_ after incubation in PBS and include dynamic piezoelectric measurements under ultrasound.

## 4 Conclusions

Overall, the findings confirm that EWP-based scaffolds are a promising, economical, and biologically relevant approach to developing advanced 3D and 4D *in vitro* CNS models.

In fact, the use of a rapid microwave-assisted synthesis based on egg white proteins and barium titanate significantly reduces production costs, manufacturing complexity, and processing time compared with conventional scaffold fabrication strategies, which often require expensive biomaterials, cross-linkers, multistep protocols, or prolonged processing conditions. Furthermore, this cost-effective approach may facilitate the reproducible and scalable production of the promising scaffolds, thereby supporting broader accessibility and translation.

The EWP:HA:BTO 6:3:1.5 formulation combines highly interconnected porosity with mechanical properties suitable for soft neural tissues, supporting efficient cell adhesion, proliferation, and early neural network formation. Scaffold polarization further enhances initial cellular attachment, highlighting the synergistic potential of structural features and surface charges. Importantly, while the present study focuses on structural, mechanical, and preliminary biological characterization, the combination of piezoelectric functionality with a dynamically responsive polymer–ceramic architecture provides a foundation for future investigations into stimulus-mediated bioelectrical activation (e.g., via ultrasound), which may enable the development of time-responsive *in vitro* systems.

## Supplementary Material









## Notes

X-ray crystallography pattern and SEM micrograph of BTO powder used as the starting piezoelectric active phase (Figure SI 1). Representative macroscopic images of each scaffold formulation (Figure SI 2) (PDF)
3D micro-CT reconstruction of the sample EWP:HA:BTO 6:3:1.5 (MP4)
Z-stack immunofluorescence video of cells cultured in DM on Day 18, showing β-III tubulin staining in red and cell nuclei in blue (MP4)
Z-stack immunofluorescence video (higher magnification) of cells cultured in DM on Day 18, showing β-III tubulin staining in red and cell nuclei in blue (MP4)
