# Supplementary material for: Stimuli-Responsive Piezoelectric Scaffold as an Advanced In Vitro Platform to Drive Neural Tissue Regeneration
Source: ACS Omega. 2026 Jul 1;11(27):40184–94. doi: 10.1021/acsomega.6c02450 (PMC13382669; doi:10.1021/acsomega.6c02450)
Supplement: Supplementary file 1 [file ao6c02450_si_001.pdf]

SUPPORTING INFORMATION

Stimuli-responsive piezoelectric scaffold as an advanced *in vitro* platform to drive neural tissue regeneration

Federica Arienti,[a,b] Noemi Ravaglia,[a,c] Giorgio Luciano,[d] Diana Pacheco,[e,f] Pietro Galizia,[a] Carlo Baldisserri,[a] Maurizio Vignolo,[d] Elisa Mercadelli,[a] Tatiana M.F. Patrício,[e,g] Sabrina Angelini,[b] Monica Montesi [a] and Silvia Panseri *[a]

[a] Institute of Science, Technology and Sustainability for Ceramics (ISSMC), National Research Council of Italy (CNR). Via Granarolo 64, 48018, Faenza, Italy
[b] Department of Pharmacy and Biotechnology, University of Bologna, Via Irnerio 48, 40126, Bologna, Italy
[c] Department of Neuroscience, Imaging and Clinical Science, University of Studies "G. D'Annunzio", 66100 Chieti, Italy
[d] Institute of Chemical Sciences and Technologies "Giulio Natta" (SCITEC), National Research Council of Italy. Via De Marini 6, 16149 Genova, Italy
[e] Centre for Rapid and Sustainable Product Development (CDRSP), Polytechnic Institute of Leiria, 2430-028 Marinha Grande, Portugal
[f] Coimbra Chemistry Centre-Institute of Molecular Sciences (CQC-IMS), Department of Chemistry, University of Coimbra, 3004-535 Coimbra, Portugal
[g] SeaPower - Association for the Development of the Sea Economy Industrial Park of Figueira da Foz R. Acácias n.º 40 – A 3090-380, Figueira da Foz, Portugal

* corresponding author, e-mail: silvia.panseri@cnr.it

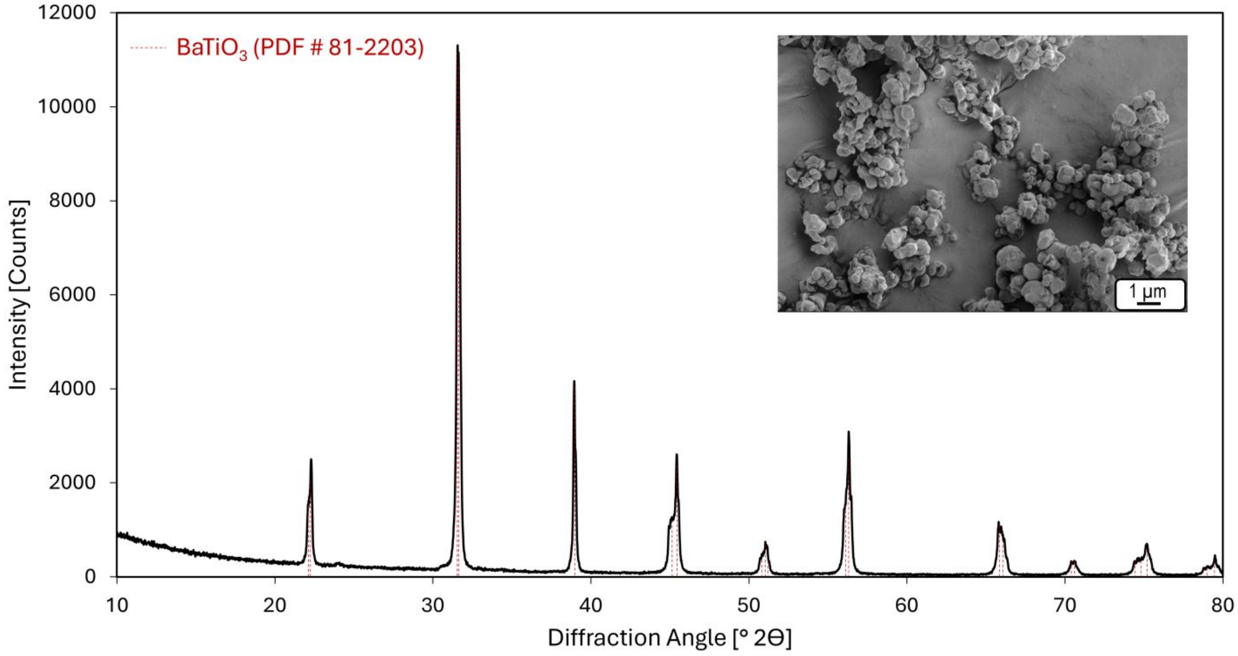

Figure SI 1. X-ray crystallography pattern and SEM micrograph of BTO powder used as starting piezoelectric active phase.

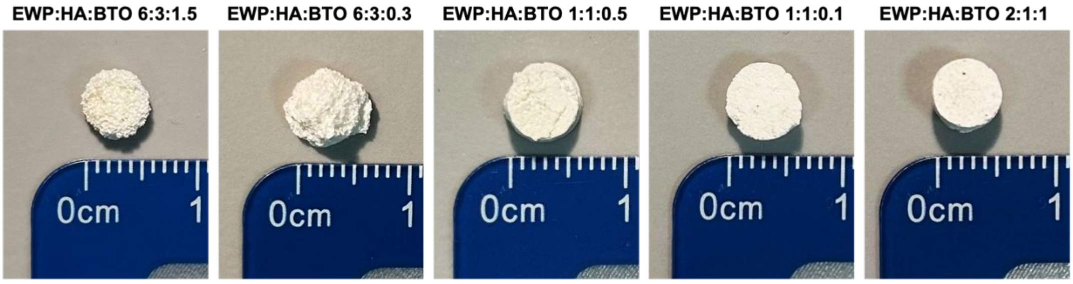

Figure SI 2. Representative macroscopic images of each scaffold formulation

Video SI 1. 3D micro-CT reconstruction of the sample EWP:HA:BTO 6:3:1.5

Video SI 2. Z-stack immunofluorescence video of cells cultured in DM at Day 18, showing β-III tubulin staining in red and cell nuclei in blue.

Video SI 3. Z-stack immunofluorescence video (higher-magnification) of cells cultured in DM at Day 18, showing β-III tubulin staining in red and cell nuclei in blue.
